# Relationship between serum phosphorus and mortality in non-dialysis chronic kidney disease patients: evidence from NHANES 2001–2018

**DOI:** 10.1186/s12882-024-03525-x

**Published:** 2024-03-06

**Authors:** Zhongcheng Fan, Rugang Li, Miaoxia Pan, Yangyang Jiang, Ying Li, Li Liu, Yang Li

**Affiliations:** 1 Department of Osteology, Haikou Municipal People’s Hospital and Central South University Xiangya Medical College Affiliated Hospital, Haikou, China; 2 Department of Nephrology, Affiliated Yuebei People’s Hospital of Shantou University Medical College, Yuebei, China; 3 Department of Nephrology, Haikou Municipal People’s Hospital and Central South University Xiangya Medical College Affiliated Hospital, 43 Renmin Ave, Haikou, 570208 China

**Keywords:** Serum phosphorus, Chronic kidney disease, Cardiovascular disease, Mortality

## Abstract

**Background:**

Hyperphosphatemia is common in chronic kidney disease (CKD), associated with higher mortality in dialysis patients. Its impact in non-dialysis patients, especially those with preserved kidney function, remains uncertain.

**Methods:**

A prospective cohort study was conducted using data from the National Health and Nutrition Examination Survey (2001–2008). Serum phosphorus was analyzed as a continuous variable, or categorized into three groups: < 3.5 mg/dL, 3.5 to < 4.5 mg/dL, and ≥ 4.5 mg/dL. Cox proportional hazards models were used to analyze the association between phosphorus with all-cause and cardiovascular disease (CVD) mortality, with or without adjustment for age, sex, race, hemoglobin, estimated glomerular filtration rate (eGFR), serum albumin, serum calcium, 25(OH)D, obesity, hypertension, diabetes, and CVD.

**Results:**

A total of 7694 participants were included in the analysis, representing 28 million CKD patients in the United States. During mean 92 months of follow up, 2708 all-cause deaths (including 969 CVD deaths) were observed. Per 1 mg/dL increase in phosphorus was associated with a 13% and 24% increased risk of all-cause mortality (hazard ratio [HR], 1.13; 95%CI, 1.02–1.24) and CVD mortality (HR, 1.24; 95%CI, 1.07–1.45), respectively. Compared with the < 3.5 mg/dL, phosphorus ≥ 4.5 mg/dL was associated with a 28% and 57% increased risk of all-cause mortality (HR, 1.28; 95%CI, 1.05–1.55) and CVD mortality (HR, 1.57; 95CI, 1.19–2.08), respectively. In participants with eGFR < 60 ml/min/1.73m^2^, elevated phosphorus (≥ 4.5 mg/ dL) were significantly associated with increased risk of all-cause mortality (HR, 1.36; 95%CI, 1.07–1.72). No significant association was observed in eGFR ≥ 60 ml/min/1.73m^2^ group (HR, 1.31; 95%CI, 0.86–1.99). This correlation does not differ significantly between subgroups defined by eGFR level (P for interaction = 0.889).

**Conclusion:**

Serum phosphorus above 4.5 mg/dL is significantly associated with a 28% and 57% increased risk of all-cause and CVD death in non-dialysis CKD patients, respectively. This relationship still demonstrated in patients with eGFR < 60 ml/min/1.73m^2^. However, for population with eGFR ≥ 60 ml/min/1.73m^2^, further verification is needed.

**Supplementary Information:**

The online version contains supplementary material available at 10.1186/s12882-024-03525-x.

## Introduction

Chronic kidney disease (CKD) is a global health issue with a prevalence of 8%-16% [1]. Early recognition and intervention of risk factors are key to improving the prognosis of CKD patients [2, 3]. In addition to recognized risk factors such as hypertension and proteinuria, research indicates that serum phosphorus can affect the prognosis of CKD by stimulating endothelial cells to cause vascular calcification [4, 5]. Elevated serum phosphorus is a significant risk factor for all-cause and CVD mortality in CKD patients on maintenance dialysis [6–8]. However, in non-dialysis CKD patients, especially those with preserved kidney function, there is less research on the correlation between serum phosphorus and mortality, and the conclusions are inconsistent. The Kidney Early Evaluation Program (KEEP) study shows that there is no association between serum phosphorus and all-cause mortality in patients with an estimated glomerular filtration rate (eGFR) < 60 ml/min/1.73m^2^ [9]. Meanwhile, the European Quality (EQUAL) study reports that serum phosphorus is independently associated with all-cause mortality in elderly patients with eGFR < 20 ml/min/1.73m^2^ [10]. For patients with an eGFR < 60 ml/min/1.73m^2^, the target for managing serum phosphorus remains inconsistent. The KDIGO guidelines suggest lowering elevated phosphorus levels toward the normal range in patients with CKD G3a-G5D (Grade 2C, 2017) [11, 12]. However, most previous studies excluded CKD patients with an eGFR ≥ 60 ml/min/1.73m^2^, so the relationship between serum phosphorus and mortality in this group of patients remains uncertain. Currently, there are no management recommendations for these patients in clinical practice [12, 13].

Given the inconsistencies in the literature, further research is needed to better describe the impact of serum phosphorus on patients with CKD, especially those with preserved kidney function. This study uses data from the National Health and Nutrition Examination Survey (NHANES) to investigate the relationship between serum phosphorus and all-cause or CVD mortality in non-dialysis CKD patients.

## Materials and methods

### Study population

NHANES is a complex, multistage sampling design, nationally representative study to assess the health and nutritional status of non-institutionalized civilians in the United States [14]. Since 1999, it has been a continuous project, with each cycle lasting two years [14]. NHANES was conducted by the Centers for Disease Control and Prevention (CDC) and and approved by the institutional review board of the National Center of Health Statistics. All participants provided written informed consent.

We used nine cycles of the NHANES from 2001 to 2018 (vitamin D data were not available in the NHANES 1999–2000). Inclusion criteria for the study were as follows: 1) age ≥ 18 years; 2) diagnosed with CKD [eGFR < 60 ml/min/1.73 m^2^ or urine albumin-to-creatinine ratio (UACR) ≥ 30 mg/g)] [13]; 3) did not start dialysis within 12 months before baseline; 4) eligible for follow up.

### Measurement of serum phosphorus

The serum phosphorus was measured using a Hitachi model 737 multichannel analyzer (Boehringer Mannheim Diagnostics, Indianapolis, IN), by observing changes in absorbance at 365 nm following phosphomolybdate formation from inorganic phosphorus in an acidic solution, which correlate directly with phosphorus levels [15].

### Assessment of covariates

Age, gender, race/ethnicity, co-medications, and comorbidities were collected from household interviews using standardized questionnaires [14]. Body weight, height were obtained when people participated in physical examinations at a mobile examination center [14]. BMI was calculated as weight in kilograms divided by height in meters squared. Race was classified as non-Hispanic White, non-Hispanic Black, Mexican American, or other. Additionally, serum calcium, creatinine, serum albumin, hemoglobin, urinary albumin, and creatinine were measured at baseline when the participants provided their blood and urine samples [14]. UACR was calculated by dividing urinary albumin by urinary creatinine [13]. eGFR values were calculated using the creatinine equation developed by the Chronic Kidney Disease Epidemiology Collaboration (CKD-EPI) [16], and CKD was staged based on eGFR according to the recommendations of the KDIGO guidelines [13], with stages 1 (eGFR ≥ 90 mL/min/1.73m^2^), 2 (eGFR 60–89 mL/min/1.73m^2^), 3a (eGFR 45–59 mL/min/1.73m^2^), 3b (eGFR 30–44 mL/min/1.73m^2^), 4 (eGFR 15–29 mL/min/1.73m^2^), or 5 (eGFR < 15 mL/min/1.73m^2^). Diabetes was defined as self-reported doctor-diagnosed diabetes, or use of insulin or oral hypoglycemic drugs, or fasting blood glucose ≥ 7.0 mmol/L, or random blood glucose ≥ 11.1 mmol/L, or glycated hemoglobin A1c (HbA1c) ≥ 6.5% [17]. Hypertension was defined as self-reported doctor-diagnosed hypertension, or systolic blood pressure ≥ 140 mmHg, or diastolic blood pressure ≥ 90 mmHg [17]. CVD included self-reported coronary heart disease, congestive heart failure, heart attack, stroke, and angina [17].

### Outcomes

Mortality from any cause and CVD was ascertained by linkage to the National Death Index through 31 December 2019. The ICD-10 was used to determine disease-specific death. CVD mortality was defined as the primary cause of death being any disease of the circulatory system (ICD-10 codes I00-I09, I11, I13, I20-I51, or I60–I69).

### Statistical analyses

Sample weights, clustering, and stratification were incorporated into all analyses because of the complex sampling design of the NHANES [14]. Participants were followed up to death or the date of 31 December 2019, whichever comes first. We take 2/9 of the 4-year weight for each individual sampled from 2001 to 2002, and 1/9 of the 2-year weight for each individual sampled from 2003 to 2018.

Serum phosphorus was analyzed as a continuous variable, or categorized into three groups: < 3.5 mg/dL, 3.5 to < 4.5 mg/dL, and ≥ 4.5 mg/dL. Baseline characteristics were summarized as means (standard errors) for continuous variables and numbers (percentages) for categorical variables. Time-to-event data were described using Kaplan–Meier curves, and the between-group difference was compared using log-rank test. The Cox proportional hazards model was used to analyze the association between serum phosphorus and all-cause or CVD mortality, with or without adjustment for confounders, including age, sex, race, hemoglobin, eGFR, serum albumin, serum calcium, 25 (OH) vitamin D, obesity, hypertension, diabetes and CVD. Additionally, a restricted cubic spline Cox regression, with the smallest Akaike information criterion (AIC) of three knots, was performed to test for linearity and explore the shape of the dose–response relation of serum phosphorus concentrations and mortality.

We further performed subgroup analysis stratified by baseline characteristics including eGFR (< 60, ≥ 60 ml/min/1.73 m^2^), sex, age (< 65, ≥ 65 years), presence/absence of CVD and obesity. We examined possible effect modification by introducing multiplicative interaction terms between phosphorus and the grouping factor into our primary Cox regression model.

Additional sensitivity analyses were conducted to verify the robustness of the results. Firstly, considering the interaction between vitamin D, blood calcium, parathyroid hormone (PTH), and serum phosphorus. We further adjusted for PTH (only provided in NHANES 2003–2006). Secondly, to reduce potential reverse causality bias, we included participants with a follow-up of more than 2 years. Thirdly, additional adjustments were conducted for co-medications (RAS inhibitors, other anti-hypertension drugs, lipid-lowering drugs, and hypoglycemic) and survey years. Finally, We made multiple imputations for missing values to avoid selection bias.

All analyses were carried out with R version 4.2.0, and a two-tailed *P* < 0.05 was considered statistically significant.

## Results

### Patient characteristics

A total of 8,363 non-dialysis CKD patients were eligible for follow up. Missing values existed among six variables. The rate of missing was 3.5%, 0.04%, 2.89%, 0.06%, 0.22% and 1.45% respectively, for BMI, hypertension, CVD, serum calcium, hemoglobin, and 25(OH) D (Details on missing data present in Supplemental Table 1). After excluding 669 participants with missing data, 7,694 participants were eventually included in the analysis (3912 UACR ≥ 3 0 mg/g only, and 3782 eGFR < 60 ml/min/1.73 m^2^ with or without UACR ≥ 30 mg/g), representing 28 million CKD patients in the United States. Among the 7694 non-dialysis CKD participants (mean age, 61 years; 46.9% male; mean eGFR, 73 ml/min/1.73m^2^), 735(10%) had high phosphorus levels (≥ 4.5 mg/dL) and 4564 (59%) between 3.5 and 4.5 mg/dL. Among these participants, 71.2%, 38.6%, and 27.6% had coexisting hypertension, diabetes, and CVD, respectively. Compared to those with the lowest phosphorus levels (< 3.5 mg/dL), patients with higher phosphorus levels were more likely to be female, non-obese (BMI < 30 kg/m^2^), and have higher CKD stages (Table 1).

**Table 1:** Baseline characteristics of patients wtih non-dialysis CKD stratified by serum phosphorus levels

**Variables** ^**a**^	**Serum phosphorus, mg/dL**			***P*** ** value**
	** < 3.5 (** ***n*** ** = 2395)**	**3.5 to < 4.5 (** ***n*** ** = 4564)**	** ≥ 4.5 (** ***n*** ** = 735)**	
Age, yr				0.083
18–65	1010(50.7)	1929(48.0)	358(52.7)	
≥ 65	1385(49.3)	2635(52.0)	377(47.3)	
Male, n (%)	1432(56.5)	1938(37.5)	241(29.7)	0.053
Race, n (%)				0.016
non-Hispanic White	1107(67.7)	2368(71.9)	371(71.1)	
non-Hispanic Black	570(13.2)	927(11.3)	146(10.5)	
Mexican American	376(8.0)	592(6.2)	108(6.8)	
Other	342(11.1)	677(10.6)	110(11.6)	
CKD stages				< 0.001
G1-2	1353(58.6)	2241(50.4)	318(44.4)	
G3a	740(30.9)	1533(34.8)	218(32.0)	
G3b	253( 8.8)	597(11.7)	111(14.5)	
G4	45(1.7)	183(3.0)	60(6.5)	
G5	4(0.1)	10(0.1)	28(2.7)	
Hemoglobin, g/dL	14.2(0.0)	13.8(0.0)	13.5(0.1)	< 0.001
Serum albumin, g/L	41.4(0.1)	41.6(0.1)	41.6(0.2)	0.152
Serum calcium, mmol/L	2.3(0.0)	2.4(0.0)	2.4(0.0)	< 0.001
Parathyroid hormone^b^, pg/mL	60.7(2.5)	53.3(1.3)	62.5(6.5)	0.023
25(OH) D, nmol/L	68.1(1.0)	70.6(0.8)	70.1(1.5)	0.054
Obesity^c^, n (%)	1080(47.4)	1910(42.3)	300(41.3)	0.004
Hypertension, n (%)	1716(68.2)	3247(66.1)	514(66.2)	0.432
Diabetes, n (%)	982(36.6)	1689(31.7)	300(34.2)	0.005
CVD, n (%)	658(24.0)	1260(24.2)	209(27.3)	0.317

*Abbreviation*: *CKD* chronic kidney disease, *CVD* cardiovascular disease

^a^All estimates accounted for complex survey designs. Continuous variables were expressed as mean (standard error). Categorical variables were expressed as number (percent)

^b^Only provided in NHANES 2003–2006 (*n* = 1601)

^c^Obesity is defined as body mass index ≥ 30 kg/m^2^

### Serum phosphorus and mortality

**Fig. 1:**
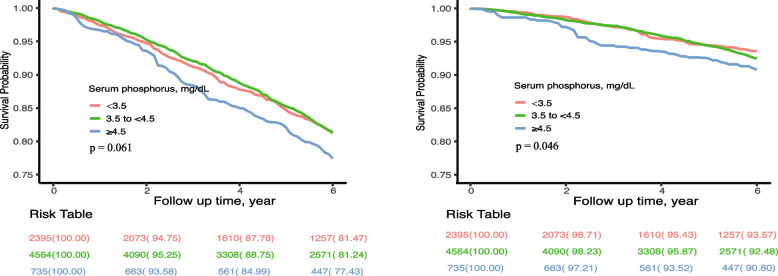
The survival probability free from all-cause (**Left**) and CVD mortality (**Right**). Abbreviation: CVD, Cardiovascular disease

**Fig. 2:**
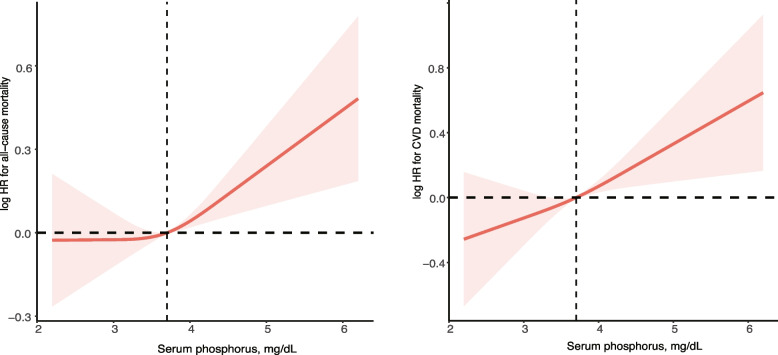
Relation of serum phosphorus concentrations with all-cause and CVD mortality. Hazard ratio (HR) was adjusted for age, sex, race, eGFR, hemoglobin, serum albumin, serum calcium, 25(OH)D, obesity, hypertension, diabetes, and CVD. Abbreviation: CVD, Cardiovascular disease; HR, hazard ratio

During a mean follow up of 92 months (median 80 [ interquartile range 43–133] months), 2708 all-cause deaths (including 969 CVD deaths) were documented. There were 811 cases (27.8%) in the 3.5 mg/ dL group, 1607 cases (29.9%) in the 3.5 to < 4.5 mg/ dL group, and 290 cases (36.3%) in the ≥ 4.5 mg/ dL group (Fig. 1). The association between serum phosphorus concentrations (Fig. 2) with the risk of all-cause and CV mortality followed a linear relationship (P for nonlinearity 0143 and 0.666). For every 1 mg/dL increase in phosphorus was associated with a 13% and 24% increased risk of all-cause mortality (hazard ratio [HR], 1.13; 95%CI, 1.02–1.24) and CVD mortality (HR, 1.24; 95%CI, 1.07–1.45), respectively (Table 2). Compared with the < 3.5 mg/dL group, phosphorus ≥ 4.5 mg/dL group was associated with a 28% and 57% increased risk of all-cause mortality (HR, 1.28; 95%CI, 1.05–1.55) and CVD mortality (HR, 1.57; 95CI, 1.19–2.08), respectively (Table 3).

**Table 2:** The association of serum phosphorus as a continuous variable with all-cause and CVD mortality

**Phosphorus, mg/dL**	**Event (rate)**	**Crude HR** **(95%CI)**	**Adjusted HR** **(95%CI)** ^**a**^	***P*** ** value**
All-cause mortality	2708(35.2)	1.09 (1.00,1.12)	1.13 (1.02,1.24)	0.016
CVD mortality	969(12.6)	1.19 (1.01,0.88)	1.24(1.07,1.45)	0.005

*Abbreviation*: *CVD* Cardiovascular disease, *HR* hazard ratio, *CI* confidence interval

^a^Adjusted for age, sex, race, eGFR, hemoglobin, serum albumin, serum calcium, 25(OH)D, obesity, hypertension, diabetes, and CVD

**Table 3:** The association of serum phosphorus levels with all-cause and CVD mortality

**Phosphorus, mg/dL**	**Event (rate)**	**Crude HR** **(95% CI)**	**Adjusted HR** **(95% CI)** ^**a**^	***P*** ** value**
**All-cause mortality**				
< 3.5	811(27.8)	Reference	Reference	-
3.5 to < 4.5	1607(29.9)	1.00 (0.89–1.13)	1.06(0.94–1.19)	0.350
≥ 4.5	290(36.3)	1.21(1.01,1.45)	1.28(1.05–1.55)	0.012
**CVD mortality**				
< 3.5	271( 9.2)	Reference	Reference	-
3.5 to < 4.5	588(10.7)	1.08(0.87–1.33)	1.19(0.95–1.50)	0.138
≥ 4.5	110(14.2)	1.43(1.09–1.88)	1.57(1.19–2.08)	0.002

*Abbreviation*: *CVD* Cardiovascular disease, *HR* hazard ratio, *CI* confidence interval

^a^Adjusted for age, sex, race, eGFR, hemoglobin, serum albumin, serum calcium, 25(OH)D, obesity, hypertension, diabetes, and CVD

**Fig. 3:**
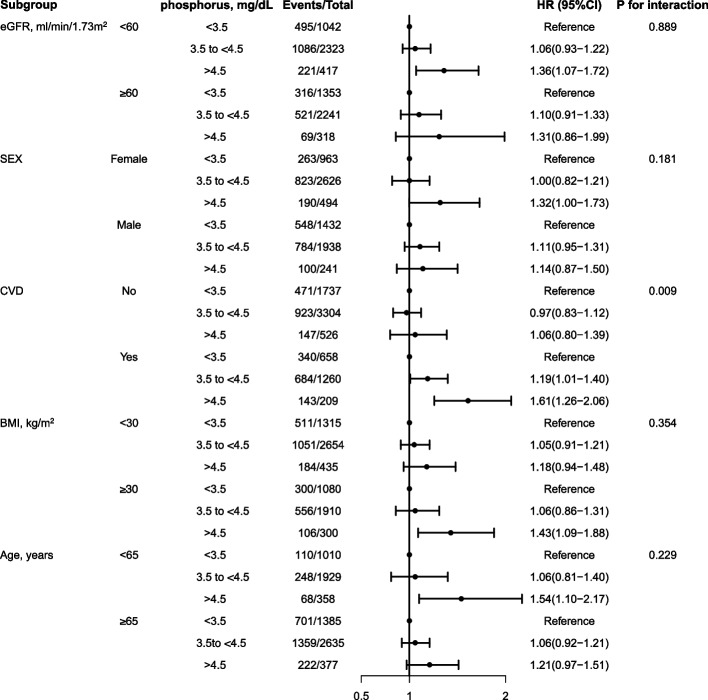
The association between serum phosphorus levels and all-cause mortality in various subgroups. Hazard ratio (HR) was adjusted for age, sex, race, eGFR, hemoglobin, serum albumin, serum calcium, 25(OH)D, obesity, hypertension, diabetes, and CVD. Abbreviation: HR, hazard ratio; CI, confidence interval; eGFR, estimated glomerular filtration rate; CVD, Cardiovascular disease

In the subgroup analysis, the association between serum phosphorus and all-cause mortality remained consistent across subgroups based on age (< 65, ≥ 65 years), sex, and obesity status. This study focused on the effects of eGFR (< 60, ≥ 60 ml/min/1.73m^2^) on serum phosphorus and mortality. In participants with eGFR < 60 ml/min/1.73m^2^, elevated phosphorus (≥ 4.5 mg/ dL) were significantly associated with increased risk of all-cause mortality (HR, 1.36; 95%CI, 1.07–1.72). Data from subgroup of eGFR ≥ 60 ml/min/1.73m^2^ did not show a significant association between elevated serum phosphorus and death (HR, 1.31; 95%CI, 0.86–1.99). However, we did not find that eGFR modifies the relationship between serum phosphorus levels and risk of all-cause death (P for interaction = 0.889). The detrimental association of hyperphosphatemia with all-cause mortality was more pronounced in the subgroup with coexisting CVD (p for interaction = 0.009) (Fig. 3).

### Sensitivity analyses

We conducted additional sensitivity analyses to validate the robustness of our results. Firstly, we further adjusted for PTH. Due to the limited number of individuals with PTH measurements (available only in NHANES 2003–2006, *n* = 1,601), the statistical power was insufficient, the association between phosphorus and all-cause mortality did not show significant differences (HR, 1.26; 95CI, 0.90–1.76). Secondly, compared with the < 3.5 mg/dL group, phosphorus ≥ 4.5 mg/dL group was associated with a 28% increased risk of all-cause mortality (HR, 1.28; 95%CI, 1.05–1.57) when we included participants with more than 2 years of follow up (*n* = 6,826) (Table 4). Our results remain stable when further adjusted for co-medications (RAS inhibitors, other anti-hypertension drugs, lipid-lowering drugs, and hypoglycemic) and survey years (Supplemental Table 2). Finally, we made multiple imputations for missing values to avoid selection bias in 8363 participants. Our result still shown that elevated serum phosphorus levels (≥ 4.5 mg/dL) was associated with a 17% increased risk of all-cause mortality (HR, 1.17; 95%CI, 1.02–1.34) (Supplemental Table 3).

**Table 4:** Sensitivity analyses for the association of serum phosphorus levels with all-cause mortality

**Serum phosphorus, mg/dL**	**Event (rate)**	**Adjusted HR** **(95% CI)**	***P*** ** value**
**Participants with PTH measurements** ^**a**^ ** (** ***n*** ** = 1601)**			
< 3.5	273(52.7)	Reference	-
3.5 to < 4.5	553(47.6)	1.06(0.89,1.28)	0.504
≥ 4.5	101(52.8)	1.26(0.90,1.76)	0.178
**Participants follow up more than 2 years** ^**b**^ ** (** ***n*** ** = 6826)**			
< 3.5	670(26.5)	Reference	-
3.5 to < 4.5	1352(28.3)	1.09(0.95,1.24)	0.226
≥ 4.5	240(33.0)	1.28(1.05,1.57)	0.015

*Abbreviation: CVD* Cardiovascular disease, *HR* hazard ratio, *CI* confidence interval, *PTH* parathyroid hormone

^a^Adjusted for PTH, age, sex, race, eGFR, hemoglobin, serum albumin, serum calcium, 25(OH)D, obesity, hypertension, diabetes, and CVD

^b^Adjusted for age, sex, race, eGFR, hemoglobin, serum albumin, serum calcium, 25(OH)D, obesity, hypertension, diabetes, and CVD

## Discussion

Our study found that elevated serum phosphorus are significantly associated with all-cause and CVD mortality in non-dialysis CKD patients. This association is consistent across subgroups stratified by eGFR (< 60, ≥ 60 ml/min/1.73m^2^), sex, obesity (no, yes), and age (< 65, ≥ 65 years). Moreover, the harmful association of elevated phosphorus with all-cause mortality is more pronounced in the population with combined CVD.

Currently, the results of studies on the relationship between serum phosphorus levels and the risk of mortality are inconsistent. The KEEP study included 10,672 non-dialysis CKD patients with eGFR < 60 ml/min/1.73m^2^. The results showed that, compared with the phosphorus < 3.3 mg/dLgroup, there was no association between serum phosphorus (levels of 3.3–3.7, 3.7–4.1, and ≥ 4.1 mg/dL) and all-cause mortality [9]. It is worth noting that although the KEEP study is large in scale, its follow up period is only 2.3 years, which may not be sufficient to capture enough endpoint events (578 patients died). In contrast, our study, which is based on a substantial number of participants and has a follow up period of 92 months, demonstrates that for every 1 mg/dL increase in serum phosphorus, the risk of all-cause mortality increases by 13%, and the risk of CVD death increases by 24%. Compared with serum phosphorus < 3.5 mg/dL, when serum phosphorus exceeds 4.5 mg/dL, it is associated with a 28% increase in the risk of all-cause mortality and a 57% increase in the risk of CVD death. Similarly, a meta-analysis of 12 cohort studies involving 25,546 non-dialysis-dependent CKD patients with 3,089 deaths (13.6%) showed that for every 1 mg/dL increase in serum phosphorus, the risk of all-cause mortality increased by 20% [18]. The EQUAL study included elderly non-dialysis CKD patients with eGFR less than 20 ml/min/1.73m^2^ from 6 centers, and the results also showed that serum phosphorus is independently associated with all-cause mortality [10]. These studies all indicate that serum phosphorus is associated with all-cause and CVD mortality in non-dialysis CKD patients.

Research shows that changes in serum phosphorus occur in the early stages of CKD and progress with the decline of eGFR [19]. However, the impact of serum phosphorus on non-renal impairment CKD patients (eGFR ≥ 60 ml/min/1.73m^2^) is still unknown [12]. Our reserch shown that elevated serum phosphorus levels (≥ 4.5 mg/dL) were significantly associated with a 36% increase in the risk of all-cause mortality in subgroup with eGFR < 60 ml/min/1.73m^2^. Although the Framingham Offspring study suggests that higher serum phosphorus levels are associated with an increased risk of CVD in individuals with eGFR ≥ 60 mL/min/1.73 m^2^ with or without proteinuria [20], our data are not conclusive. However, we also have no evidence that this association differs significantly between subgroups defined by eGFR levels (< 60, ≥ 60 ml/min/1.73m^2^), suggesting that changes in serum phosphorus may require attention in the early CKD stages. In patients with early CKD, this relationship needs to be validated by subsequent studies involving larger populations.

Furthermore, elevated phosphorus levels are associated with a greater risk of all-cause mortality in individuals with CVD. Compared with serum phosphorus < 3.5 mg/dL, serum phosphorus levels of 3.5 to < 4.5 and ≥ 4.5 mg/dL are associated with a 19% and 61% increase in all-cause mortality risk, respectively. Therefore, more attention should be paid to phosphorus levels in CKD patients with CVD [21].

This is a nationally representative sample survey study, and weighted statistics were used, making the conclusions reliable and highly generalizable. In addition, unlike previous studies that mainly focused on dialysis or advanced CKD patients, our analysis covers all stages of CKD, especially in the population with eGFR ≥ 60 ml/min/1.73m^2^, which is rarely included in previous studies. We still found that hyperphosphatemia may increase patient mortality risk. Considering the mutual influence of elevated serum phosphorus on serum calcium, vitamin D, and PTH metabolism [12], we conducted additional analyses. After adjusting for serum calcium, vitamin D, and PTH as confounding factors, the results still showed that elevated serum phosphorus is independently associated with an increased risk of all-cause mortality in CKD patients, further supporting our conclusions. This study also has limitations. Firstly, the NHANES database mainly collects data from the US population. It should be cautious when generalizing the finding to other ethnics. Secondly, our results rely on a single serum phosphorus measurement, rather than repeated measurements. However, previous research has shown that repeated measurements of serum phosphorus may improve exposure estimation and thus strengthen the association with mortality rate [22]. Thirdly, despite our extensive adjustment for confounding factors, there may still be significant unmeasured confounders. Finally, recognizing the critical role of serum phosphorus in the mortality risk of CKD patients, therapies to reduce serum phosphorus are expected to reduce the mortality risk of these patients. However, our study design cannot further clarify this issue.

In conclusion, the results of this large-sample cohort study indicate that serum phosphorus above 4.5 mg/dL is associated with an increased risk of all-cause and CVD mortality in non-dialysis CKD patients. This relationship still demonstrated in patients with eGFR < 60 ml/min/1.73m^2^. However, for population with eGFR ≥ 60 ml/min/1.73m^2^, further verification is needed.

### Supplementary Information

**Supplementary Material 1.**

## Data Availability

The datasets generated and analyzed in the current study are available at NHANES website: https://www.cdc.gov/nchs/nhanes/index.htm.
