# Supplementary material for: Relationship between serum phosphorus and mortality in non-dialysis chronic kidney disease patients: evidence from NHANES 2001–2018
Source: BMC Nephrol. 2024 Mar 6;25:89. doi: 10.1186/s12882-024-03525-x (PMC10918918; doi:10.1186/s12882-024-03525-x)
Supplement: Supplementary file 1 — Supplementary Material 1. [file 12882_2024_3525_MOESM1_ESM.docx]

**Relationship between serum phosphorus and mortality in non-dialysis chronic kidney disease patients: evidence from NHANES 2001-2018**

**SUPPLEMENTAL MATERIAL**

**Table of Contents**

**Supplemental Table 1. Baseline characteristics of included and excluded patients**

**Supplemental Table 2.** **Sensitivity analyses for the association of serum phosphorus levels with all-cause mortality by further adjusting co-medications and survey years**

**Supplemental Table 3.** **The association of serum phosphorus levels with all-cause mortality in participants by multiple imputations for missing values (n=8363)**

**Supplemental Table 1.** **Baseline characteristics of included and excluded patients**

| **Variables ^a^** | **Included**  **(n=** **7694)** | **Excluded**  **(n=669)** |  |
| --- | --- | --- | --- |
| Age, yr |  |  | <0.001 |
| 18-65 | 3297(49.3) | 386(59.9) |  |
| ≥65 | 4397(50.7) | 283(40.1) |  |
| Male, n (%) | 3611(42.4) | 292(43.8) | 0.575 |
| Race, n (%) |  |  | <0.001 |
| non-Hispanic White | 3846(70.5) | 293(63.8) |  |
| non-Hispanic Black | 1643(11.8) | 135(10.6) |  |
| Mexican American | 1076( 6.8) | 141(10.4) |  |
| Other | 1129(10.8) | 100(15.2) |  |
| CKD stages |  |  | <0.001 |
| G1-2 | 3912(50.8) | 427(63.8) |  |
| G3a | 2491(33.3) | 138(23.7) |  |
| G3b | 961(11.1) | 79(11.6) |  |
| G4 | 288(2.9) | 23(3.7) |  |
| G5 | 42(0.4) | 2(0.1) |  |
| Hemoglobin, g/dL | 13.9(0.0) | 13.8(0.1) | 0.438 |
| Serum albumin, g/L | 41.5(0.1) | 41.2(0.3) | 0.286 |
| Serum calcium, mmol/L | 2.4(0.0) | 2.4(0.0) | 0.871 |
| 25(OH) D, nmol/L | 69.8(0.7) | 67.2(2.2) | 0.239 |
| Obesity ^b^, n (%) | 3290(43.7) | 94(26.2) | <0.001 |
| Hypertension, n (%) | 5477(66.7) | 335(54.7) | <0.001 |
| Diabetes, n (%) | 2971(33.4) | 192(29.4) | 0.133 |
| CVD, n (%) | 2127(24.5) | 156(32.3) | 0.005 |

^a^ All estimates accounted for complex survey designs. Continuous variables were expressed as mean (standard error). Categorical variables were expressed as number (percent).

^b^ Obesity is defined as body mass index≥30kg/m^2^.

**Abbreviation**: CKD, chronic kidney disease; CVD, cardiovascular disease.

**Supplemental Table 2. Sensitivity analyses for the association of serum phosphorus levels with all-cause mortality by further adjusting co-medications and survey years**

| **Serum phosphorus, mg/dL** | **Adjusted HR ^a^**  **(95% CI)** | **P value** |
| --- | --- | --- |
| < 3.5 | Reference | - |
| 3.5 to <4.5 | 1.07(0.96,1.20) | 0.237 |
| ≥ 4.5 | 1.30(1.07,1.57) | 0.008 |

^a^ Adjusted for age, sex, race, eGFR, hemoglobin, serum albumin, serum calcium, 25(OH)D, obesity, hypertension, diabetes, CVD, co-medications (RAS inhibitors, other anti-hypertension drugs, lipid-lowering drugs, and hypoglycemic), and survey years.

**Abbreviation**: CVD, Cardiovascular disease; HR, hazard ratio; CI, confidence interval.

**Supplemental Table 3.** **The association of serum phosphorus levels with all-cause mortality in participants by multiple imputations for missing values (n=8363)**

| **Serum phosphorus, mg/dL** | **Event (rate)** | **Adjusted HR ^a^**  **(95% CI)** | **P value** |
| --- | --- | --- | --- |
| < 3.5 | 904 (35.2) | Reference | - |
| 3.5 to <4.5 | 1766 (35.6) | 1.04(0.95,1.13) | 0.393 |
| ≥ 4.5 | 312 (37.6) | 1.17 (1.02,1.34) | 0.021 |

^a^ Adjusted for age, sex, race, eGFR, hemoglobin, serum albumin, serum calcium, 25(OH)D, obesity, hypertension, diabetes, CVD, co-medications (RAS inhibitors, other anti-hypertension drugs, lipid-lowering drugs, and hypoglycemic), and survey years.

**Abbreviation**: CVD, Cardiovascular disease; HR, hazard ratio; CI, confidence interval.
